# Endophytes: a uniquely tailored source of potential antibiotic adjuvants

**DOI:** 10.1007/s00203-024-03891-y

**Published:** 2024-04-06

**Authors:** Ashaimaa Y. Moussa

**Affiliations:** Department of Pharmacognosy, Faculty of Pharmacy, Ain-Shams University, African Union Organization Street, Abbassia, Cairo, 11566 Egypt

**Keywords:** Endophytes, Microbial resistance, Antibiotic adjuvants

## Abstract

Multidrug microbial resistance is risking an annual loss of more than 10 million people’ lives by 2050. Solutions include the rational use of antibiotics and the use of drugs that reduce resistance or completely obliterate them. Here endophytes come to play due to their high-yield production and inherent nature to produce antimicrobial molecules. Around 40%, 45% and 17% of antibacterial agents were obtained from fungi, actinomycetes, and bacteria, respectively, whose secondary metabolites revealed effectiveness against resistant microbes such as MRSA, MRSE, and *Shigella flexneri*. Endophyte’s role was not confined to bactericidal effect but extended to other mechanisms against MDR microbes, among which was the adjuvant role or the “magic bullets". Scarce focus was given to antibiotic adjuvants, and many laboratories today just screen for the antimicrobial activity without considering combinations with traditional antibiotics, which means real loss of promising resistance combating molecules. While some examples of synthetic adjuvants were introduced in the last decade, the number is still far from covering the disused antibiotics and restoring them back to clinical use. The data compiled in this article demonstrated the significance of quorum sensing as a foreseen mechanism for adjuvants from endophytes secondary metabolites, which call for urgent in-depth studies of their molecular mechanisms. This review, comprehensively and for the first time, sheds light on the significance of endophytes secondary metabolites in solving AMR problem as AB adjuvants.

**Supplementary Information:**

The online version contains supplementary material available at 10.1007/s00203-024-03891-y.

## Introduction

Resistance to antibiotics is a hundred-year-old dilemma in the medical field and no less dangerous than pandemics like the corona virus, with an estimated more than 1 trillion $ healthcare expenses in 2050 (WHO 2019; Antimicrobial resistance collaborators 2022). Since the advent of the first historical antibiotic penicillin, discovered in 1930 by Alexander Fleming, bacterial evading mechanisms commenced to be developed soon after. The WHO chief Tedros Adhanom Ghebreyesus announced, "It threatens to unwind a century of medical progress and leaves us defenseless against infections that once were treated easily". The three reasons behind bacterial resistance are the limited accessibility of antimicrobial agents to inside the cells or active efflux pumps, the expression of inactivating enzymes targeting antimicrobial molecules, biofilm formation and the absence of a drug target (Darby et al. 2023; Azevedo et al. 2015)**.** The epidemics encountered were dominated by resistant microbes, which included but were not limited to methicillin-resistant *Staphylococcus aureus* (MRSA), ciprofloxacin-resistant strains, extensively resistant tuberculosis (XDR-TB), and vancomycin-resistant enterococci (VRE) with a reported degree of resistance exceeding 97%, 92.9%, 52%, and 59% for these strains, respectively (Centers for disease control and prevention 2019; Neil et al. 2016). This and the lack of effective treatment exacerbated the health care system; furthermore, the more MDR spreads, the more virulent it becomes with the propensity to transfer horizontally to other strains (Larsson et al. 2022; Moussa et al. 2023).

More importantly, new approaches were highly considered as antibiotic adjuvants to circumvent microbial resistance mechanisms. These molecules show sub-inhibitory antimicrobial effect; thus, they are not subjected to selection pressure by bacteria, which leads to activity restoration and conservation of antibiotics. Plants-based secondary metabolites were promising as source of antibiotic adjuvant molecules. Being cheap, and relatively safe, particularly in Third World countries, plants presented several effective remedies to human pathogens. (Phanstiel et al. 2019; Khameneh et al. 2019). Yet, plants were limited in their availability and chemical diversity compared to fungi (Hutchings et al. 2020). Here in, secondary metabolites from fungal and bacterial endophytes come into play (Pasrija et al. 2022; Kemkuignou et al. 2021; Melander et al. 2020).

Endophytes are microbial species inhabiting in symbiotic or mutualistic relationship different parts of plants as roots, seeds, flowers, fruits, stems and leaves without causing apparent harm (Abdel Razek et al. 2019; Alanzi et al. 2023, AbdelRazek et al. 2023). Endophytes co-evolved with plants since the beginning of their land existence and carried out several of their traits as secondary metabolite production and their fight against environmental stresses and attacking pathogens (Kealey et al. 2017). Many of these pathogens need colonization and QS to secure their ecological niches and to develop AMR; consequently, endophytes were naturally adapted to hamper this cross talk through many ways. Some endophytes produce enzymes like acylases and lactonases; others evolved to produce chemicals, which act by quorum quenching to suppress microbial pathogenesis (Pellissier et al. 2021). Biofilm inhibition was seen in several endophytes with a rare distribution that signals directed co-evolution of endophytic microbes to harbor quorum quenching molecules as the 1,3,6-trihydroxy-7-methoxy-9H-xanthen-9-on isolated from the halophyte associated fungi *Penicillium citrinum*-314 (Abdel Razek et al. 2020). This compound was noted to be produced simultaneously in two endophytic species within the same extreme inhabiting host, *Halocnemum strobilaceum* grown in Egyptian marshes and manifested a 100% reduction in biofilm formation. The combination therapy approach can exploit endophyte-derived compounds to inhibit resistance genes through their synergistic action with traditional antibiotics. Even though they may not be superior if administered alone, the endophyte-based adjuvant molecules can reduce the net MIC values and preserve the antibiotic reserve (Khameneh et al. 2019; Dhanda et al. 2023). Upon viewing the literature, we intensely felt the need to write this review, which aims to introduce the role of endophytic non-microbicidal secondary metabolites as a (game changer) in the antibiotic resistance war when used in combination with obsolete antibiotics. This innovative approach supported by the current biotechnological know-hows could solve the microbial resistance problem and restore the golden era of antibiotics. Examples of promising antibiotic adjuvants were listed from endophytes showing their potency in rescuing bacterial susceptibility by folds magnitude.

## Mechanisms of antibiotic adjuvants

Antibiotic adjuvants are non-microbicidal compounds that will render bacteria weaker and enhance effectiveness of the antimicrobial agents, among which are molecules that inhibit microbial adaptive responses and resistance. Quorum quenching is one of the successful mechanisms to stop both Gram-positive and Gram-negative microbial cellular communication leaving them alive with attenuated virulence, which suppress the microbial tendency to develop more resistance (Naik et al. 2021). The cellular efflux of antibiotics is another mechanism to limit the concentration of the used antimicrobial agents inside the cell. Compounds that block the activity of efflux pumps EP (EPIs) through inhibiting gene expression or impeding pump elements assembly are highly sought-after. Another welcomed supplement in the antibiotic adjuvant toolbox is the membrane permeability enhancers. Antibiotics need to diffuse through cell membranes and outer membranes (OM) in case of Gram-negative bacteria; for instance, NV716 can bind to OM *Pseudomonas aeruginosa* and re-sensitize it to obsolete antibiotics such as chloramphenicol, and doxorubicin (Wang et al. 2021). A short overview on the available antibiotic adjuvants would encompass class I adjuvants, with β-lactamase inhibitors as the only approved example, which work through inhibiting different resistance modes as efflux pumps, inactivating proteins, or essential targets (Friis et al. 1991) (Wright et al. 2016). β-Lactamase inhibitors directly act on serine B-lactamases to block their activity, thus restore B-lactam core antibiotics. For instance, clavulanic acid was first introduced in 1976 and widely marketed combined with amoxicillin as augmentin, yet it was not effective against Class A, C, and D carbapenemases, which hydrolyze cephalosporins, monobactam, penicillins, meropenem and imipenem (Carcione et al. 2021). The FDA approved Avycaz was a combination of the adjuvant avibactam and ceftazidime that was effective against Types A, C, and D serine β-lactamases, yet not showing any activity against Class C and D carbapenemases (Alfei et al. 2022). Another DBO-based adjuvant, relebactam was introduced with imipenem as combination in 2019 with no activity against class D carbapenems. (Bhagunde et al. 2019). Enzyme inactivating adjuvants such as tropolones, which inhibit adenylyl transferases, and pyrazolopyrimidines selectively acting on microbial aminoglycoside kinase aminoglycoside 3'-phosphotransferase APH(3′)-I, were other good examples despite their limited activity in vivo (Feder et al.^2008^).

Similarly, gentamycin activity was boosted by 6-furanylquinazolines by blocking the aminoglycoside 2-O nucleotidyl transferase 2″-Ia. Wortmannin, a phosphatidylinositol 3-kinase inhibitor, was one of the pioneer natural products that blocked aminoglycoside kinases and displayed covalent alternation of the lysine in the ATP binding pocket (Boehr et al. 2001). Another unique mechanism of antibiotic adjuvants is the efflux pump inhibition since most of the resistance mechanism depend on the bacterial efflux activity whether it encompass the inner membrane, the periplasm, the LPS outer membrane or spanning the three layers. While reserpine and celecoxib rescued fluoroquinolones activity by inhibiting the NorA protein belonging to the major facilitator superfamily (MFS) of *S. aureus* (Sabatini et al. 2012), celecoxib only enhanced drugs as chloramphenicol, kanamycin and ampicillin in MRSA. Other adjuvants mechanisms were identified with their synergistic effect with antibiotics through unclear genetic or physiological factors. These were discovered by cell-based assays as resistance breakers, for example, teichoic acid synthesis inhibitors. Wall teichoic acid formed mainly of 1–5 linked ribitol-5-phosphate units in *S. aureus* caused B-lactamase resistance in MRSA because it catalyzed the PBP2a function (Sewell et al.^2014^).

Cefuroxime was potentiated again (FIC is 0.040) when combined with ticlopidine due to its TarO inhibitory effect against clinical MRSA isolates. In the same way, tunicamycin inhibits TarO, which is involved in teichoic acid biosynthesis could reduce oxacillin MIC dramatically to 0.4 μg/mL (Farha et al. 2013).

**Fig. 1:**
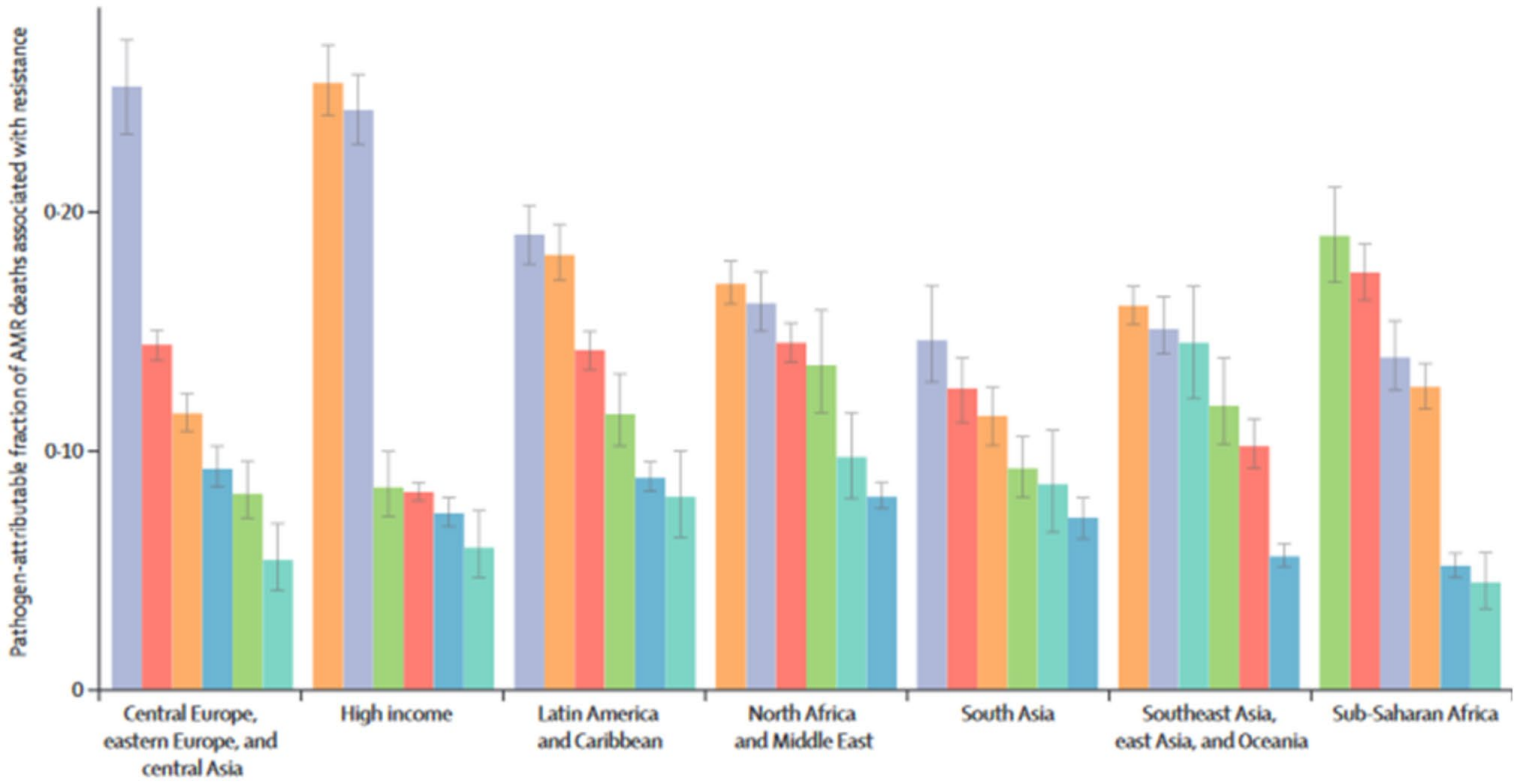
Compounds from endophytes with promising anti-virulence mechanisms (Cont). Fraction of deaths attributed to AMR of the six leading resistance bacteria, from left to right (*A. baumannii, E. coli, K. pneumonia, P. aeruginosa, S. aureus, and S. pneumonia)* (Antimicrobial resistance, 2022)

Other membrane-targeting adjuvants were characterized as class 1b adjuvants, which mostly included antimicrobial peptides as polymyxin-derived analogues. These compounds were repurposed against Gram-negative bacteria. For instance, PMBN, PMBO and PMBH were known for their potentiation of Gram-positive antibiotics when combined with teicoplanin or vancomycin (Dhanda et al. 2019). Furthermore, SPR741 revived clarithromycin and fusidic acid by disrupting the outer membrane structure with a reduction up to 8000-fold in MIC against and *K. pneumonia and E. coli.* Venturicidin A was an ATP synthase inhibitor isolated from soil actinomycetes and succeed in stalling the proton flow in the ATP synthase; thus, rescued gentamycin activity to FIC 0.64 in MRSA and *P. aeruginosa* (Dhanda et al. 2023) (Fig. 1).

## Methodology

A thorough investigation of literature was conducted using PubMed, PubChem, ScienceDirect, Web of Science, Google Scholar, and Scopus. The inclusion criteria were adjusted as follows: time period from 1980 to 2023, source of the adjuvant: endophyte fungi or bacteria, compounds isolated: at least one compound active as antibiotic adjuvant, source of endophyte: different parts of terrestrial plants or its rhizosphere. Several key words were employed in the search with Boolean operator criteria as “endophytes antibiotic adjuvants”, endophytes secondary metabolites antimicrobial”, “endophytes NMR adjuvants”, “endophytes quorum sensing inhibitors NMR”, “endophytes efflux pump inhibitors NMR”, and “endophytes and biofilm inhibitor compounds”. Exclusion criteria were total extract activity only, lack of isolated compounds, source of bioactive compound is a non-endophytic microbe or marine source. Initially, search results provided 40,700 articles, which were narrowed down to 2600 based on the mentioned criteria. Further manual inspection yielded 100 articles after prioritizing high cite scores and significance of results.

## Current scenario of antibiotic resistance

Multidrug microbial resistance has created a vicious cycle with more and more virulence developed in resistant strains and transferred to others by horizontal gene transfer (Davies et al. 2010). Additionally, traditional treatment regimens with obsolete antibiotics available at cheap prices over the counter demanded the discovery of game changers. Not only human pathogens are involved in MDR, but also veterinary pathogens and zoonotic bacteria with more than 200,000 tons of annual antibiotic production dedicated to agriculture and veterinary medicine (Laxminarayan et al. 2013). While in the USA and Europe around 35,000 deaths were reported, Africa and Southwest Asia infection percentage was around 6 and 18%, respectively (WHO 2019). India and many other third world countries encountered deep uncontrolled usage of antibiotics as well as pollution cases in food and air, which supported the escalation of resistant infections (Tagliaferri et al. 2020). Cefiderocol was the only introduced antibiotic in a five-year period until 2021 to treat Pseudomonas aeruginosa, Acinetobacter baumannii, and Enterobacteriaceae (Wang et al.^2022^). According to the WHO reports, the number of antibiotics in clinical trials pipelines were only 27 compared to 1300 anticancer drugs in 2020 (Butler et al. 2023). The list of deleterious pathogens included not only bacteria, but also fungi as Candida auris, whose mortality rate exceeded 40% within 90 days (Mishra et al. 2023). Resistance mechanisms to drug molecules differs according to the type of bacteria, their intrinsic or acquired resistance, which may be one of the following: active drug efflux (2) inactivating a drug; (3) limiting drug entry to the cell or (4) changing the drug target. Gram positive or negative bacteria follow different attitudes.

### Gram-positive organisms

Gram-positive bacteria cannot limit drug access to the cells because it lacks the outer membrane and rarely use drug efflux strategies (Linciano et al. 2019). Other mechanisms could be to change the structure or the number of (penicillin-binding proteins) PBPs, which are the proteins involved in peptidoglycan formation. VISA strains of *S. aureus* developed an unclear strategy to form a thicker wall, which caused vancomycin resistance in Gram-positive bacteria (Lamber 2002). Aminoglycosides are polar molecules thus they are difficult to penetrate the hydrophobic cell wall in enterococci while in mycobacteria, fluoroquinolones and rifampicin are hydrophobic enough to enter easily (Kumar et al. 2005).

### Gram-negative organisms

In addition to drug efflux pumps, the lipopolysaccharide layer in the outer membrane of Gram-negative bacteria is the best-known intrinsic resistance mechanism for example: ampicillin, glycopeptides are ineffective against Acinetobacter spp., and Escherichia coli are resistant to macrolides. (Reygaert 2018). Moreover, daptomycin displays cell membrane depolarization effect and the glycopeptide vancomycin constrains the cell wall synthesis of Gram-negative bacteria (Randall et al. 2013), and represent a major factor in the development of the (VRE—vancomycin-resistant enterococci). Similarly, in *Staphylococcus aureus (MRSA)*, the van genes acquired resulted in the structural changes of peptidoglycans and accordingly reduced binding affinity of vancomycin. Larger molecules of antibiotics also have a lower chance to pass through the LPS layer of Gram-negative bacteria.

Acquired resistance can be due to mutations in key regulatory genes of drug targets, drug transporters or regulators are lethal to bacterial cells despite being of low percentage, 1 for every 10^6^ to 10^9^ cell divisions. Porin channels control the hydrophilic drug uptake into the cells with large outer membrane usually through reductions in their number and/or modified selectivity (Blair et al. 2014). This was observed in the resistance of Neisseria gonorrhoeae to tetracycline and β-lactams and Enterobacteriaceae to carbapenems (Thiolas et al. 2004; Reygaert 2018). Gene mutations could alter cellular structures or cause enzymatic modifications; thus, reducing drug or efficacy. Daptomycin resistance was due to the mprF gene mutation, which rendered the cell membrane surface positive and prohibited calcium binding (Stefani et al. 2015). Fluoroquinolones bactericidal effect through inhibition of nucleic acid synthesis was eliminated when bacteria acquired modifications in topoisomerase IV in Gram-positive or DNA gyrase in Gram-negative bacteria. This mutation obviated the ability of the drug to bind to these proteins (Redgrave et al. 2014). Another resistance mechanism is drug degradation as in β-lactamases or in tetracycline where tetX gene induced hydrolysis and subsequent inactivation (Reygaert 2018). Adenylation or phosphorylation in aminoglycosides inactivated the drugs and inhibited binding; in the same way, acylation was found to be dominant in chloramphenicol, and acetylation in fluoroquinolones and streptogramins (Zhang et al. 2017b).

## Endophytes as an untapped source of antibiotic adjuvants

Endophytes are unique since they colonize plants’ inner tissues and protect them from diseases. More than any other natural sources, endophytes are the factory of a wide range of bioactive metabolites such as alkaloids, phenolic acids, steroids, quinones, terpenoids, saponins, and tannins (Kemkuignou et al. 2022). This chemical diversity meant higher potential to act as anti-inflammatory, immunomodulating, antimalarial, antiviral, antidiabetic, anti-arthritis, and anti-tuberculosis agents (Fadiji et al. 2020). Fungal non-microbicidal molecules are to be used in combination with antibiotics to strengthen their activity (Estrela et al. 2016).

Therefore, drug discovery based on fungi-derived antimicrobial adjuvants would be an auspicious approach, which will promote a better antibiotic regimen in clinical use. Many compounds are screened but show weaker activity that does not lead them to further advancement in clinical pipelines. These need to be studied in combination regimens with obsolete antibiotics rather than being excluded. A strategy that will even limit further genetic mutations and resistance (Zacchino et al. 2017, Moussa et al. 2022). An example of combination therapies is trimethoprim and sulfa drugs, cefuroxime and ticlopidine whose additive effect was superior against MRSA; moreover, β-lactamase inhibitors as clavulanate, sulbactam and tazobactam were potent in their activity when combined with amoxicillin, ampicillin and piperacillin, respectively (Huttner et al. 2020).

## Secondary metabolites from endophytic bacteria

**Fig. 2:**
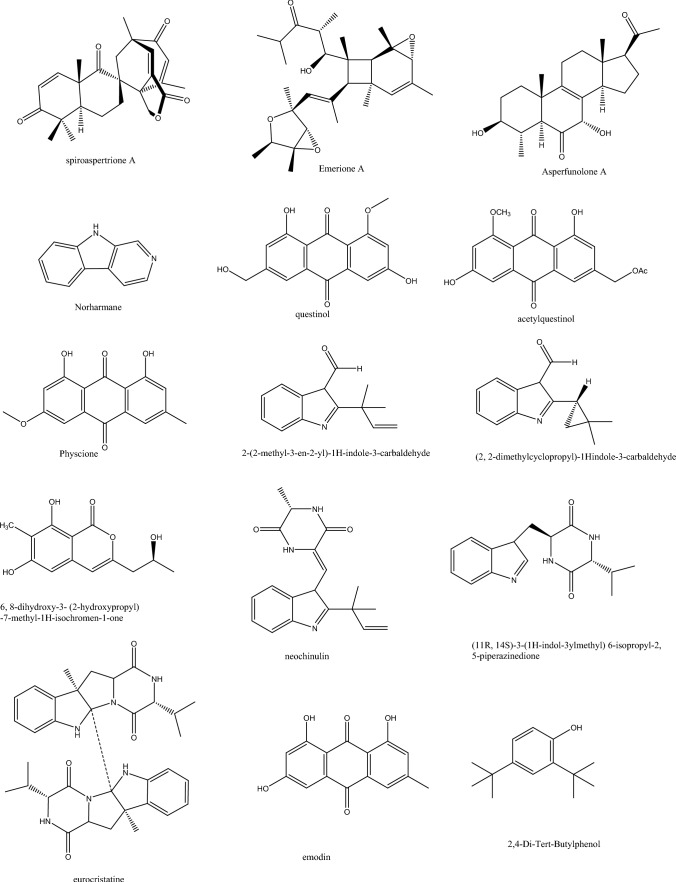
Compounds from endophytes with promising anti-virulence mechanisms (cont.)

Linear dipeptides as proline–glycine and N-amido-α-proline detected by ESI-MS/MS from the marine sponge-derived *Streptomyces* sp. was shown to inhibit QS in *P. aeruginosa* at a concentration of 0·1 mg/mL. Similarly, cinnamic acid was detected and revealed a more significant QS inhibitory effect than a 1:1 ratio of Pro-Gly dipeptide: cinnamic acid mixture (Elbondkly et al. 2021; Sumi et al. 2015). The endophyte *Streptomyces cyaneochromogenes* RC1 was the source of actinomycin D, which loosened biofilm structures and weakened motility and other virulence factors in *P. aeruginosa* PAO1. Real-time PCR indicated the downregulation of major QS gene expression as *pilA*, *pqsR*, *lasI*, *rhlI, pslA, and rhlR* and the dramatic reduction in signaling metabolites of N-butanoyl-L-homoserine lactone and N-(3-oxododecanoyl)-L-homoserine lactone (Zeng et al. 2022). The uropathogen *Serratia marcescens* was found to be susceptible to gentamicin after its combination with phenol, 2,4-bis(1,1-dimethylethyl). This phenolic compound was isolated from *Vibrio alginolyticus* G16 and manifested a significant reduction in biofilm (85%) together with several QS enzymes such as extracellular polysaccharide (84.62%), protease (41.9%), prodigiosin (84.5%), hemolysin (69.9%), and lipase (84.3%). Likewise, this was confirmed by qPCR and lowering of biofilm genes *bsmA, flhD, fimA, and fimC* [Padmavathi et al. 2014; Chen et al. 2019) (see Table 1, Fig. 2).

**Table 1:** Endophytic compounds with their host, fungal source, and anti-virulence effects

	Compounds	Source	Endophyte	Target	Biology	References
*Molecules showing B-lactam antibiotic synergism*						
1	Emerione A	*Hypericum perforatum leaves*	*Aspergillus sp. TJ23*	*Anti- New Delhi metal-β-lactamase 1 (NDM-1)*	NMD-1 inhibitory activity, IC50 = 12.1 ± 0.9 μM. Restored activity of meropenem against *E.coli and K. pneumoniae* ATCC BAA-2146	Yan et al. (2022)
2	Aspergillomarasmine A	*Hordeum vulgare L.*	*Pyrenophora teres*	*anti-metallo B-lactamases (B1, B2, and B3)*	rescue meropenem *in vitro* and *in vivo*	King et al. (2014)
3	Asperfunolone A	*Hypericum perforatum leaves*	*Aspergillus sp. TJ23*	*Anti- New Delhi metal-β-lactamase 1 (NDM-1)*	NMD-1 inhibitory activity	Yan et al. (2022)
*Molecules showing vancomycin synergism*						
4	Questinol	*Twig of Rhizophora mucronata Poir*	*Eurotium chevalieri KUFA 0006*	*NA*	Anti-biofilm activity of *E.coli*. Synergistic association with vancomycin against the multidrug-resistant VRE B3/101	Melander et al. (2020); May Zin et al. (2017)
5	Acetylquestinol	*Twig of Rhizophora mucronata Poir*	*Eurotium chevalieri KUFA 0006*	*NA*	Anti-biofilm activity of *E.coli* synergistic association with vancomycin against the multidrug-resistant VRE *E. faecali*s B3/101	Melander et al. (2020); May Zin et al. (2017)
6	Physcion	*Twig of Rhizophora mucronata Poir*	*Eurotium chevalieri KUFA 0006*	*NA*	Anti-biofilm of S. aureus and *Enterococcus faecalis*. Synergistic association with vancomycin against the multidrug-resistant VRE *E. faecalis* B3/101	Melander et al. (2020); May Zin et al. (2017)
7	2-(2-Methyl-3-en-2-yl)-1H-indole-3-carbaldehyde	*Twig of Rhizophora mucronata Poir*	*Eurotium chevalieri KUFA 0006*	*NA*	Inhibition of biofilm production in *S. aureus* ATCC 25923. Synergistic association with vancomycin against the multidrug-resistant VRE B3/101	Melander et al. (2020); May Zin et al. (2017)
8	(2, 2-Dimethylcyclopropyl)-1H indole-3-carbaldehyde	*Twig of Rhizophora mucronata Poir*	*Eurotium chevalieri KUFA 0006*	*NA*	Significant reduction in biofilm production by *E. coli* ATCC 25922. Inhibition of biofilm production in *S. aureu*s ATCC 25923. Synergistic association with vancomycin against the multidrug-resistant VRE *E. faecalis* B3/101	Melander et al. (2020); May Zin et al. (2017)
9	6, 8-Dihydroxy-3- (2-hydroxypropyl)-7-methyl-1H-isochromen-1-one	*Twig of Rhizophora mucronata Poir*	*Eurotium chevalieri KUFA 0006*	*NA*	Significant reduction in biofilm production by *E. coli* ATCC 25922. Inhibition of biofilm Production in *S. aureus* ATCC 25923. Synergistic association with vancomycin against the multidrug-resistant VRE B3/101	Melander et al. 2020); May Zin et al. (2017
10	Neochinulin	*Twig of Rhizophora mucronata Poir*	*Eurotium chevalieri KUFA 0006*	*NA*	Significant reduction in biofilm production by E. coli ATCC 25922. inhibition of biofilm Production in S. aureus ATCC 25923. synergistic association with vancomycin against the multidrug-resistant VRE B3/101	Melander et al. (2020); May Zin et al. (2017)
11	(11R, 14S)-3-(1H-Indol-3ylmethyl) 6-isopropyl-2, 5-piperazinedione	*Twig of Rhizophora mucronata Poir*	*Eurotium chevalieri KUFA 0006*	*NA*	Significant reduction in biofilm production by E. coli ATCC 25922. inhibition of biofilm Production in S. aureus ATCC 25923. synergistic association with vancomycin against the multidrug-resistant VRE E. faecalis B3/101	Melander et al. (2020); May Zin et al. (2017)
12	Eurocristatine	*Twig of Rhizophora mucronata Poir*	*Eurotium chevalieri KUFA 0012*	*NA*	Significant reduction in biofilm production by *E. coli* ATCC 25922. inhibition of biofilm Production in S. aureus ATCC 25923. synergistic association with vancomycin against the multidrug-resistant VRE *E. faecalis* B3/101	Melander et al. (2020); May Zin et al. (2017)
*Molecules showing ampicillin synergism*						
13	Emodin	*Twig of Rhizophora mucronata Poir*	*Eurotium chevalieri KUFA 0006*	*NA*	Synergistic activity with oxacillin towards MRSA *Staphylococcus aureus*	May Zin et al. (2017)
14	Spiroaspertrione A	*Hypericum perforatum leaves*	*Aspergillus sp. TJ23*	*Inhibition of penicillin-binding protein 2a (PBP2a)*	Reduced the oxacillin MIC to 1 µg/mL towards MRSA and inhibited PBP2a expression	Yan et al. (2017)
15	2,4-Di-tert-butylphenol	*Tridax procumbens leaves*	*Daldinia eschscholtzii*	*1.Reduced the secretion of virulence factors, 2. reduced the expression of quorum sensing-related genes, i.e., lasI, lasR, rhlI, and rhlR, 3.restricted the adhesion and invasion of P. aeruginosa to the A549 cancer cell line*	Synergism with ampicillin against *P. aeruginosa*. Inhibit receptors *LasR and RhlR* in *P. aeruginosa*. Both a standalone and an antibiotic adjuvant	Mishra et al. 2020); Viszwapriya et al. (2016)
*Molecules showing aminoglycosides synergism*						
16	Phenol, 2,4-bis(1,1-dimethylethyl)	*Seaweed Gracilaria gracilis*	*Vibrio alginolyticus G16*	*Reduction of virulence factors and EPS up to 84%*	Reduce biofilm formation in *Serratia marcescens*, revealed a synergistic effect with gentamicin	Padmavathi et al. (2014); Chen et al. (2019)
17	Venturicidin A	*Scindapsus hederaceus, Shorea ovalis and Zingiber71 spectabile*	*Streptomyces SUK 10*	*Uncoupling of ATP synthesis*	Gentamycin potentiation	Yarlagadda et al. (2020)
18	Shikonin	*Roots of Lithospermum officinale*	*Fusarium tricinctum*	*Inhibit the protein expression related to drug resistance and S. aureus exotoxins*	Reduced the MIC of streptomycin by up to 16-fold. Active against *S. aureus* RN4220 with MIC of 16 mg/L	Mone et al. (2021); Mollaei et al. (2019); Li et al. (2022)
*Molecules showing cephalosporins synergism*						
19	Chitosan	*Several plant sources*	*Aspergillus flavus*	*Reduction in lasR and rhlR genes expression*	Showed synergism with ceftazidime with a reduction in MIC from 128 to 64 μg/ml. Reduced biofilm formation of *P. aeruginosa*	Muslim et al. (2018)
*QS and biofilm inhibitors (unidentified synergism)*						
20	(5S,6S)-6-((3’S,4’S,Z)-3’,4’-Dihydroxypent-1-en-1-yl)-5-hydroxy-5,6-dihydro-2H-pyran-2-one	*Tropical palm Astrocaryum sciophilum*	*Lasiodiplodia venezuelensis*	*Reduced the expression of lasB and rhlA*	Highly efficient in lowering the production of rhamnolipids’ level as a virulence factor	Pellissier et al. (2021)
21	(Z)-3-((2R,3R,6R)-3-Hydroxy-6-((R)-1-hydroxyethyl)-3,6-dihydro-2H-pyran-2-yl)acrylamide	*Tropical palm Astrocaryum sciophilum*	*Lasiodiplodia venezuelensis*	*Reduced the expression of lasB and rhlA*	Moderate lowering of the production of rhamnolipids’ level and its precursors	Pellissier et al. (2021
22	Sphaeropsidin A	*Forest plants*	*Diplodia corticola*	*NA*	Inhibited biofilm formation of MRSA and *P. aeruginosa* MDR	Pompilio et al. (2023); Roscetto et al. (2020)
23	Vulculic acid	*Taxus baccata*	*Chaetosphaeronema achilleae*	*NA*	Inhibited biofilm formation by *S. aureus* at 256 μg/mL	Narmani et al. (2019); Pompilio et al. (2023)
24	Curvulol	*Taxus baccata*	*Chaetosphaeronema achilleae*	*NA*	Inhibited biofilm formation by *S. aureus* at 256 μg/mL	Narmani et al. (2019); Pompilio et al. (2023)
25	Sclerin	*Trunk of Fagus sp.*	*Hypoxylon fragiforme,*	*NA*	Biofilm formation of the pathogen *S. aureus*, inhibited by 86%	Yuyama et al. (2017;
26	Sclerin diacid	*Trunk of Fagus sp.*	*Hypoxylon fragiforme,*	*NA*	Inhibition 80% of the biofilm of *S. aureus*	Yuyama et al. (2017);
27	Cytochalasin A	*Forest collection*	*Hypoxylon fragiforme*	*NA*	Inhibited 91% biofilm formation by *S. aureus*	Yuyama et al. 2018
28	Phenochalasins A	*Decaying wood, Thailand forest*	*Daldinia eschscholtzii*	*NA*	Inhibited biofilm formation by *S. aureus*	Yuyama et al. (2018
29	Phenochalasins B	*Decaying wood, Thailand forest*	*Daldinia eschscholtzii*	*NA*	Inhibited biofilm formation by *S. aureus*	Yuyama et al. (2018)
30	Chaetoglobosin A	*Decaying wood, Thailand forest*	*Ijuhya vitellina*	*NA*	Inhibited biofilm formation by *S. aureus *(87.3%)	Yuyama et al. (2018)
31	Cytochalasin C	*Decaying wood, Thailand forest*	*Metarrhizium anisopliae*	*NA*	Inhibited biofilm formation by *S. aureus* (42%)	Yuyama et al. (2018)
32	L-696,474	*Decaying wood, Thailand forest*	*Hypoxylon fragiforme*	*NA*	Inhibited biofilm formation by *S. aureus* (44%)	Yuyama et al. (2018)
33	19,20-Epoxycytochalasin C	*Decaying wood, Thailand forest*	*Rosellinia rickii*	*NA*	Inhibited biofilm formation by *S. aureus *(40%)	Yuyama et al. (2018)
34	Phenochalasin D	*Decaying wood, Thailand forest*	*Hypoxylon cf. kretzschmarioides BBH 42276*	*NA*	Inhibited biofilm formation by *S. aureus *(43%)	Yuyama et al. (2018)
35	Thioketopiperazine 1	*Glycyrrhiza glabra*	*Phoma sp.*	*Inhibited staphyloxanthin production in S. aureus (32.4%), inhibited bacterial transcription/translation (70.0%)*	Inhibiting the virulence and growth of *S. aureus*	Arora et al. (2016)
36	Thioketopiperazine 2	*Glycyrrhiza glabra*	*Phoma sp.*	*Inhibited staphyloxanthin production in S. aureus (42.1%), inhibited bacterial transcription/translation (90%)*	Inhibiting the virulence and growth of *S. aureus*	Arora et al. (2016)
37	Actinomycin D	*Areca catechu L*	*Streptomyces cyaneochromogenes RC1*	*inhibition of motility and virulence factors as rhamnolipid, pyocyanin, siderophores, and protease*	Reduced the virulence factors and biofilm formation of *P. aeruginosa* PAO1	Zeng et al. (2022)
38	1-(4-Amino-2-hydroxyphenyl)ethanone	*Leaves of Punica granatum*	*Phomopsis liquidambari S47*	*Reduced the secretion of acyl-homoserine lactones*	Attenuated the virulence of *P. aeruginosa* PAO1	Zhou et al. (2021)
39	Mixture of F.A: palmitic, arachidic, stearic, oleic, and linolenic fatty acids	*Coriandrum sativum leaves*	*Arthrographis kalrae*	Inhibited water insoluble EPS production	Inhibition of *S. mutans* biofilms at conc of 31 μg/mL	Abdel-Aziz et al. (2020)
40	Nor-harmane	*Leaves of Orychophragmus violaceus*	*Irpex lacteus*	*Reduced fimbriae production*	Suppressed *S. marcescens NJ01* virulence factors	Luo et al. (2021); Lee et al. (2017)
41	Wortmannin	*Inner tissues of Aloe vera*	*Talaromyces wortmannii*	*PI3-kinase inhibitor*	Suppressed *B. anthracis* infection of A549 cells	King et al. (2014)

The dental bacteria *Streptococcus mutans* biofilm formation was disrupted by an array of fatty acids isolated from the endophyte *Arthrographis kalrae* where unsaturated, mainly linoleic and oleic acids, and saturated components represented 93.8% and 5.8% of the total analyzed fraction, respectively. Full biofilm inhibition was shown by concentration 31.3 mg/mL, and a direct correlation was signified between the extracellular matrix and the biofilm inhibitory effect (Abdel Aziz et al. 2020).

## Secondary metabolites from endophytic fungi

Oxacillin activity against MRSA (ATCC43300) was rescued in combination with spiroaspertrione A, the polyketide spiro meroterpenoid whose precursor andiconin B was inactive. While the former terpenoid revealed an MIC value of 4μg/mL via inhibiting PBP2a in MRSA (ATCC43300) (He et al. 2017), it reduced the MIC of oxacillin by 32-fold from 32 µg/mL to 1 µg/mL. This re-sensitization puts forward the use of spiroaspertrione A and other polyketide–terpene hybrids as possible adjuvants for antibiotics.

Following in silico screening, the highly methylated polyketide emerione A isolated from *Aspergillus* sp. TJ23 restored the activity of the β-lactam antibiotic meropenem and carbapenem towards New Delhi metal-β-lactamase 1 (NDM-1) producing strains of family Enterobacteriaceae (He et al. 2022). The structure activity study revealed binding to the zinc ion inside NDM-1 active center. Carbapenem-resistant Enterobacteriaceae (CRE) are among the most critical virulent resistance because of their broad spectrum and challenging treatment. Both *K. pneumoniae* ATCC BAA-2146 and NDM-1-producing *E. coli* strains were rendered susceptible after treatment of emerione A (He et al. 2022). Nor-harmane demonstrated an anti-biofilm effect against *P. aeruginosa PA14, Klebsiella oxytoca, E. coli O157: H7, and P. aeruginosa PAO1 (*Khan et al. 2017). The same alkaloid was isolated from *Irpex lacteus* fungus and showed anti-QS and anti-biofilm effect towards *S. marcescens *(Luo et al. 2021; Lee et al. 2017). Not only were biofilm genes downregulated as manifested by the qPCR, but also nor-harmane restored sensitivity to ofloxacin. Additionally, the FT-IR revealed reduction of various metabolites of the extracellular matrix.

*Eurotium chevalieri KUFA 0006* was isolated from the mangrove *Rhizophora mucronata* Poir yielding diketopiperazine and polyhydroxy anthraquinones as well as anthranilic acid, isochromone and prenylated indole 3-carbaldehyde derivatives (May Zin et al. 2017). While emodin augmented oxacillin effect against MRSA 66/1, it was bactericidal towards Gram-positive (*E. faecalis* ATCC 29212 and *S. aureus* ATCC 25923) and Gram-negative (*P. aeruginosa* ATCC27853 and *E. coli* ATCC 25922). Emodin, physcion and the two new prenylated indole carbaldehydes inhibited biofilm formation of *S. aureus* ATCC 25923, and others inhibited biofilm formation of *E. coli* ATCC 25922. Moreover, all of them were synergistic with vancomycin against multidrug-resistant VRE B3/101 (Zhang et al. 2014; May Zin et al. 2017)** (**Table 1**).**

**Fig. 3:**
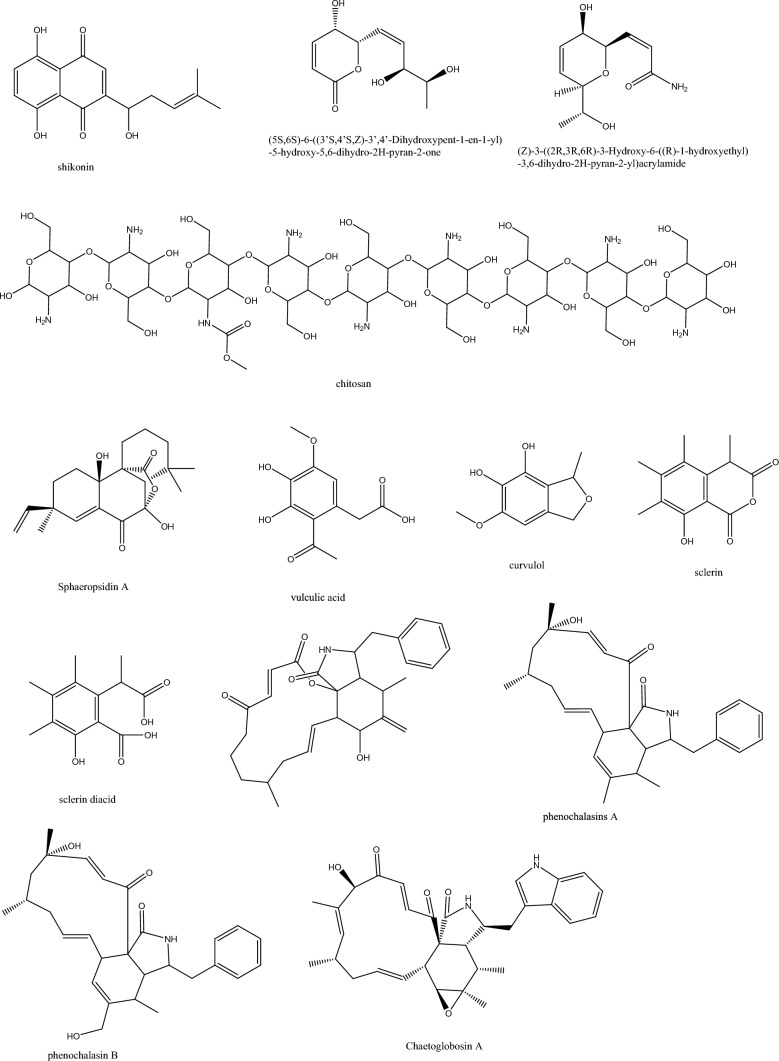
Compounds from endophytes with promising anti-virulence mechanisms (cont.)

From the endophytic *Daldinia* sp., the wood inhabiting tropical fungi, 2,4-di-tert-butylphenol (2,4-DBP) was isolated and showed anti-biofilm formation and anti-QS activity towards *P. aeruginosa.* This was in accordance with previous reports that showed 2, 4-DBP role in biofilm reduction of *S. pyogenes* as well as in restricting surface hydrophobicity, EPS production and bacterial adhesion (Mishra et al. 2020). While in *C. albicans* 2,4-DBP both restricted the formation and disrupted the preformed biofilms, it altered polysaccharides, proteins, and EPS components in *Serratia marcescens* (Viszwapriya et al. 2016). 1-(4-amino-2-hydroxyphenyl) ethanone (AHE) suppressed acyl-homoserine lactones and QS-related genes; moreover, it inhibited antioxidant enzymes and subsequently enhanced oxidative stress in *P. aeruginosa* PAO1. 1,4-Naphthoquinones are remarkable in their antibacterial, antifungal or antiparasitic activities even though the structural features might differ from one molecule to another (Zhou et al. 2021) Among the susceptible pathogens from the Gram-positive group is *Staphylococcus* spp.-representative bacterium of the ESKAPE pathogens, and from the Gram-negative group are *Pseudomonas* spp. (9%) and *E. coli* (11%). The plausible mechanisms of action for 1,4-naphthoquiones are inhibition of ROS, suppression of Eps, and curing plasmids elimination (Mishra et al. 2020). From the endophytic fungus *Fusarium tricinctum*, the naphthoquinone shikonin was isolated in reasonable amounts to facilitate its introduction to the industrial scale (Li et al. 2022) (Fig. 3). Shikonin revealed a synergistic activity with seven conventional antibiotics, namely, oxacillin, chloramphenicol, gentamicin, ceftazidime, amikacin, amoxicillin, and cefoxitin using the Checkerboard method against MRSA. The postulated mechanisms involved *S. aureus* biofilm and virulence factors inhibition, interfering with the cell membrane integrity and expression of PBP2a (Mollaei et al. 2019).

Chitosan or the deacetylated chitin, the linear polysaccharide obtained from insects’ cuticle, algae and fungi, was isolated in a yield of 53.8% from the endophytic *Aspergillus flavus* through a biological method of bacterial demineralization and deproteination (Muslim et al. 2018)*.* Chitosan synergistically enhanced the antimicrobial potential of ceftazidime, which decreased its MIC values from 1024 and 512 μg/mL to 128 and 64 μg/mL against *P. aeruginosa and S. aureus*, respectively The substituted pyran derivatives **19**.and **20** with the former comprising an α, β-unsaturated δ-lactone were isolated from the fungus *Lasiodiplodia venezuelensis* colonizing the ancient tropical palm trees and manifested anti-QS effect by reduction of *lasB and rhlA* expression and impaired key virulence factors without harming. *P. aeruginosa* growth (Pellissier et al. 2021). The pyrone scaffold was reported in several studies to possess an antimicrobial effect because of their role as Michael acceptors. For instance, phomopsolides from the endophytic *Penicillium sp*. displayed feeding deterrent effects for Scolytid beetles (Li et al. 2022; Mone et al. 2021; Stierle et al. 1997).

The diterpenoid sphaeropsidin A was isolated from the terrestrial endophyte *Diplodia corticola,* and its synergistic activity in combination with epi-epoformin was assessed revealing a reduction in MIC values down to ¼ MIC regarding sphaerospidin A and 1/16 MIC regarding epi-epoformin against Gram-negative bacteria. The same combination was evaluated towards Gram-positive bacteria and showed lower MIC values of sphaerospidin A (1/2 MIC) and epi-epoformin (1/32 MIC) (Pompilio et al. 2023; Roscetto et al. 2020). This adjuvant role of sphaerospidin A was safer to the cells, with a dose of 3.12 μg/mL for each of them and conferred cellular viability of 60%, as evidently shown. After measuring the fractional inhibitory concentration (FIC), an additive effect was noted with Gram-positive bacteria (FIC > 0.5) and synergistic effect with Gram-negative (FIC < 0.5). Moreover, biofilm formation of *P. aeruginosa* clinical and reference strains was inhibited by 62% and 50%, and that of MRSA strains was inhibited by 53% and 60%, respectively (Roscetto et al. 2020). The phenolic derivatives, vulculic acid and curvulol showed nearly the same inhibition of biofilm formation against *S. aureus* by 96.82% at 256 µg/mL while their MIC values were around 33.33 μg/mL towards *S. aureus* DSM 346 and *Bacillus subtilis* DSM 10 (Dobretsov et al. 2011; Narmani et al. 2019).

**Fig. 4:**
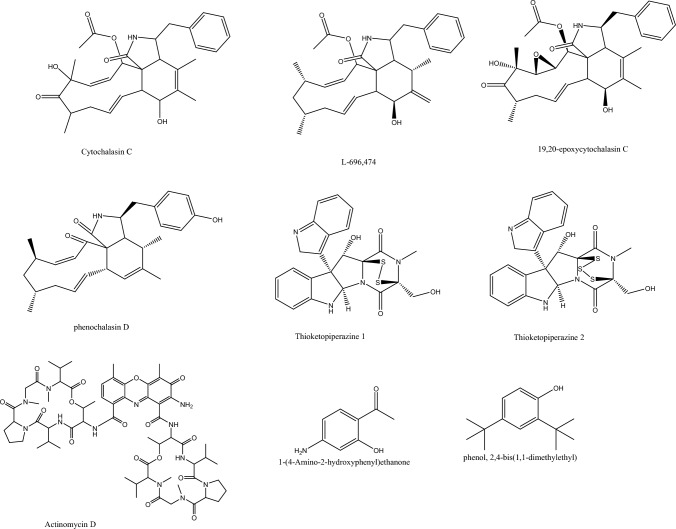
Compounds from endophytes with promising anti-virulence mechanisms (cont.)

*Hypoxylon fragiforme*, the beech wood-associated fungus, was endowed with high chemical diversity based on its developmental stages and fermentation conditions, which was clearly manifested in the previously reported cytochalasins and azaphilones production (Li et al. 2003). Sclerin and sclerin diacid from *Hypoxylon* sp. revealed weak or no antibacterial activity while their anti-biofilm activity against *S. aureus* was highly specific and up to 85% inhibition. It seemed that biofilm inhibition provided a crucial benefit to fungi in restricting bacterial growth and thus outcompeting them in the biological niche because even the change of media composition of *Hypoxylon* stimulated the biosynthesis of other biofilm inhibiting compounds (Yuyama et al. 2017; Stadler et al. 2006). Moreover, cytochalasans, known for their weak antibacterial effect, uncommonly inhibited biofilm formation up to 90% (Yuyama et al. 2018). A pair of thio-diketopiperazines were isolated from *Phoma* sp. with about 10μM MIC value against *S. aureus and Streptococcus pyogenes*. Both thio-derivatives enhanced streptomycin activity with an anti-biofilm effect. Furthermore, they regulated the pathogenicity genes in at sub-lethal concentration in *S. aureus*; thus, they were good potential candidates as antibiotic adjuvants (Yuyama et al. 2018). The strategy of using antibiotic adjuvants is quite attractive because you can activate many antibiotics with a single molecule (Kong et al. 2017). The unique biological niches tapped by endophytes highly propose promising antimicrobial bioactivities that facilitate life in this ecology (Fig. 4). Aspergillomarasmine A, the polycarboxylic amino compound first isolated from *Aspergillus versicolor* (Handayani et al. 2023) was recorded to influence Zn^+2^ availability and undermine bacterial survival. Similarly, it inhibited NDM-1 receptor to halt antibiotic resistance (Sychantha et al. 2021; Zhang et al. 2017a) (Pieri et al. 2014). The fungal-derived aspergillomarasmine A (King et al. 2014; Rotondo et al. 2020) was directed towards the carbapenem-resistant Gram-negative pathogens *Enterobacteriaceae, Pseudomonas spp and Acinetobacter spp*.by inhibiting the NMD-1 enzyme. Stigmasterol is a phytosterol of tetracyclic triterpenoidal structure widely biosynthesized in plants and was recorded from the endophytes of Nigerian plants to greatly enhance the β-lactam antibiotics as ampicillin against resistant strains by 98.7% reduction in colony count (Yenn et al. 2017; Ibrahim et al.^2021^).

Fatty acids identified in the endophytic *Arthrographis kalrae* obtained from *Coriandrum sativum were* active against the virulence factors *of Streptococcus mutans,* the major producer of biofilm and extracellular matrix. A mixture of unsaturated and saturated fatty acids was regarded effective in suppressing the EPS of *S. mutans* at a concentration of 31.3 μg/mL (Abdel Aziz et al. 2021).

Wortmannin is a PI3-kinase inhibitor that was repurposed to act as antibiotic adjuvant and exerted a promising role against *B. anthracis* infection of A549 cells (Bara et al. 2013) (Fleeman 2023). PI3-K is among the virulence factors or bacterial targets that help their replication, colonization; thus, escaping the bactericidal effect of antimicrobials (Xu et al. 2010).

Venturicidin A was isolated from an endophytic actinomycete and displayed a potentiation effect when given with the aminoglycoside gentamycin against multidrug-resistant clinical isolates of Enterococcus, Staphylococcus, and Pseudomonas aeruginosa (Yarlagadda et al. 2020) with a fast eradication rate of MRSA. This was attributed to the venturicidin A activity in blocking the ATP synthesis from electron transport, consequently, raising the proton concentration outside the cell and facilitating more gentamycin uptake with a concomitant membrane dysregulation mode.

## Future perspectives

During the previous decades, natural products discovery of antibacterial leads markedly declined because of high throughput screening, isolation costs, and combinatorial chemistry libraries, which were not natural product friendly. Endophytes and soil microbes are the limitless source of bioactive antimicrobial discovered till now. Despite natural products chemical diversity, research was confined to only screening approaches. To resolve the feebleness of drug discovery, promising entities from both bacterial and fungal endophytes are sought on the other side, these molecules are better investigated as antibiotic adjuvants if not showing significant antimicrobial effect. This will act to complement synergistic combinations provided that the molecules demonstrated sufficient safety, eliminate infections, prevent future evolutionary mutations, and eradicate antibiotic resistance. It is worth noting that the concept of antibiotic adjuvants from endophytes will open the way to one molecule-multiple combinations approach, which is the best to address the antibiotic crisis.

### Supplementary Information

Below is the link to the electronic supplementary material.

Supplementary file1 (DOCX 14 KB)

## Data Availability

Not applicable.
