# Supplementary material for: Endophytes: a uniquely tailored source of potential antibiotic adjuvants
Source: Arch Microbiol. 2024 Apr 6;206(5):207. doi: 10.1007/s00203-024-03891-y (PMC10998792; doi:10.1007/s00203-024-03891-y)
Supplement: Supplementary file 1 — Supplementary file1 (DOCX 14 KB) [file 203_2024_3891_MOESM1_ESM.docx]

The author would like to thank the reviewers for their valuable time and comments and appreciate their constructive addition to this work. All changes were highlighted in red in the manuscript.

Reviewer 5

The authors have followed the instructions suggested to improve the quality of the manuscript.
They have added the references and have changed the manuscript quality in particular section. I hope they have taken the help from a native English speaker.
It could be accepted now.

Response: Thanks very much for your effort

Reviewer 1

The author has responded to all comments in a proper way. Some minor comments have been found:
comment: Add a figure legend to figure 1.

Response: done per your suggestion

comment: Italicize S. aureus in line 198.

Response: done and thanks very much for your precise revision.

comment: Add “.” At the end of each paragraph after the references not before.

Response: done
